# The complete chloroplast genome sequence of *Gentiana atropurpurea* and phylogenetic analysis

**DOI:** 10.1080/23802359.2021.1875931

**Published:** 2021-02-17

**Authors:** Chen Liu, Zhen Hou

**Affiliations:** a Laboratory of Histology and Cytology, Hunan Agen Medicine Laboratory Technology Co., Ltd, Changsha, PR China; b Institute of Environmental Biology, Faculty of Science, Utrecht University, Utrecht, The Netherlands; c China West Normal University, Nanchong, Sichuan, PR China

**Keywords:** *Gentiana atropurpurea*, chloroplast genome, phylogenetic analysis

## Abstract

*Gentiana atropurpurea* is an annual herb belonging to section Microsperma T.N. Ho series Suborbisepalae Marquand. This species is endemic to China with its distribution limited to the southeast of the QTP. In this study, the complete chloroplast genome sequence of *G. atropurpurea* was characterized from Illumina pair-end sequencing. The chloroplast genome of *G. atropurpurea* was 145,757 bp in length, containing a large single-copy region (LSC) of 78,287 bp, a small single-copy region (SSC) of 16,750 bp, and two inverted repeat (IR) regions of 25,360 bp. The overall GC content is 37.90%, while the corresponding values of the LSC, SSC, and IR regions are 35.8, 31.7, and 43.4%, respectively. The genome contains 132 complete genes, including 86 protein-coding genes (62 protein-coding gene species), 37 *tRNA* genes (29 *tRNA* species), and eight *rRNA* genes (four rRNA species). Phylogenetic analysis based on complete chloroplast genomes showed that *G. atropurpurea* and *G. tongolensis* clustered together as sisters to other related species.

*Gentiana atropurpurea* T. N. Ho, which is an annual herb belonging to section Microsperma T.N. Ho series Suborbisepalae Marquand. This species is endemic to China with its distribution limited to the southeast of the QTP. *Gentiana atropurpurea* has been a poorly studied species within a large genus, except for its classification and taxonomy. *Gentiana atropurpurea* represents an excellent model for understanding how different evolutionary forces have sculpted the variation patterns in the genome during the process of population differentiation and ecological speciation (Neale and Antoine 2011). Here, we report heteroplasmy in the plastome of *G. atropurpurea* at the nucleotide level. This information will help botanists to reconsider the heredity and evolution of chloroplasts and to take caution with their use as genetic markers.

Fresh and clean leave materials of *G. atropurpurea* were collected from Daocheng, Sichuan Province in the QTP (28°52′E, 100°16′N) in Sichuan province, China and quickly dried with silica gel for DNA extraction. Meanwhile, the voucher specimens with flowers (HZLD001) were collected and deposited at the Herbarium of Laboratory of Histology and Cytology, Hunan Agen Medicine Laboratory Technology Co., Ltd. Total genomic DNA was extracted from fresh leaves by using the improved CTAB method (Doyle 1987). The obtained DNA was fragmented to construct a paired-end library with an insert-size of 350 bp, and the genome sequencing was performed using Illumina HiSeq 2000 (Novogene, Tianjin, China) platform. The raw sequence data has been deposited into the Genome Sequence Archive at the BIG Data Center, Beijing Institute of Genomics (BIG), Chinese Academy of Sciences, under accession numbers CRR055506. The raw data was filtered using Trimmomatic version .0.32 with default settings. The output was a 1.4 Gb raw data of 150 bp paired-end reads. We used the software MITObim version 1.8 (Hahn et al. 2013) and metaSPAdes (Nurk et al. 2017) to assemble chloroplast genomes with the cp genome of closely related species *Gentiana obconica* (NC037981) as the reference. Finally, we annotated the chloroplast genome using the software DOGMA (Wyman et al. 2004) with the cp genome of *G. obconica* (NC037981) as the reference genome, and then corrected the results using Geneious version 8.0.2 (Campos et al. 2016) and Sequin version 15.50 (http://www.ncbi.nlm.nih.gov/Sequin/).

The annotated chloroplast genomes of *G. atropurpurea* were submitted to the GenBank under the accession number of MT593367. The chloroplast genome of *G. atropurpurea* was 145,757 bp in length, containing a large single-copy region (LSC) of 78,287 bp, a small single-copy region (SSC) of 16,750 bp, and two inverted repeat (IR) regions of 25,360 bp. The overall GC content is 37.90%, while the corresponding values of the LSC, SSC, and IR regions are 35.8, 31.7, and 43.4%, respectively. The genome contains 132 complete genes, including 86 protein-coding genes (62 protein-coding gene species), 37 *tRNA* genes (29 *tRNA* species), and eight *rRNA* genes (four rRNA species).To further investigate its taxonomic status, a maximum-likelihood (ML) tree was constructed based on complete chloroplast genome sequences using MEGA version 7.0 (Kumar et al. 2016) with 1000 replicates. The program operating parameters were set as follows: a Tamura 3-parameter (T92) nucleotide substitution model with 1000 bootstrap repetitions, accompanied by Gamma distributed with Invariant site (G + I) rates, and partial deletion of gaps/missing data. We used the complete chloroplast genomes sequence of *G. atropurpurea* and 22 other related species to construct phylogenetic tree. The 23 chloroplast genome sequences were aligned with MAFFT (Katoh and Standley 2013), and then the ML tree was constructed (Figure 1). The phylogenetic analysis revealed that *G. atropurpurea* and *Gentiana tongolensis* clustered together as sisters to other related species.

**Figure 1.**
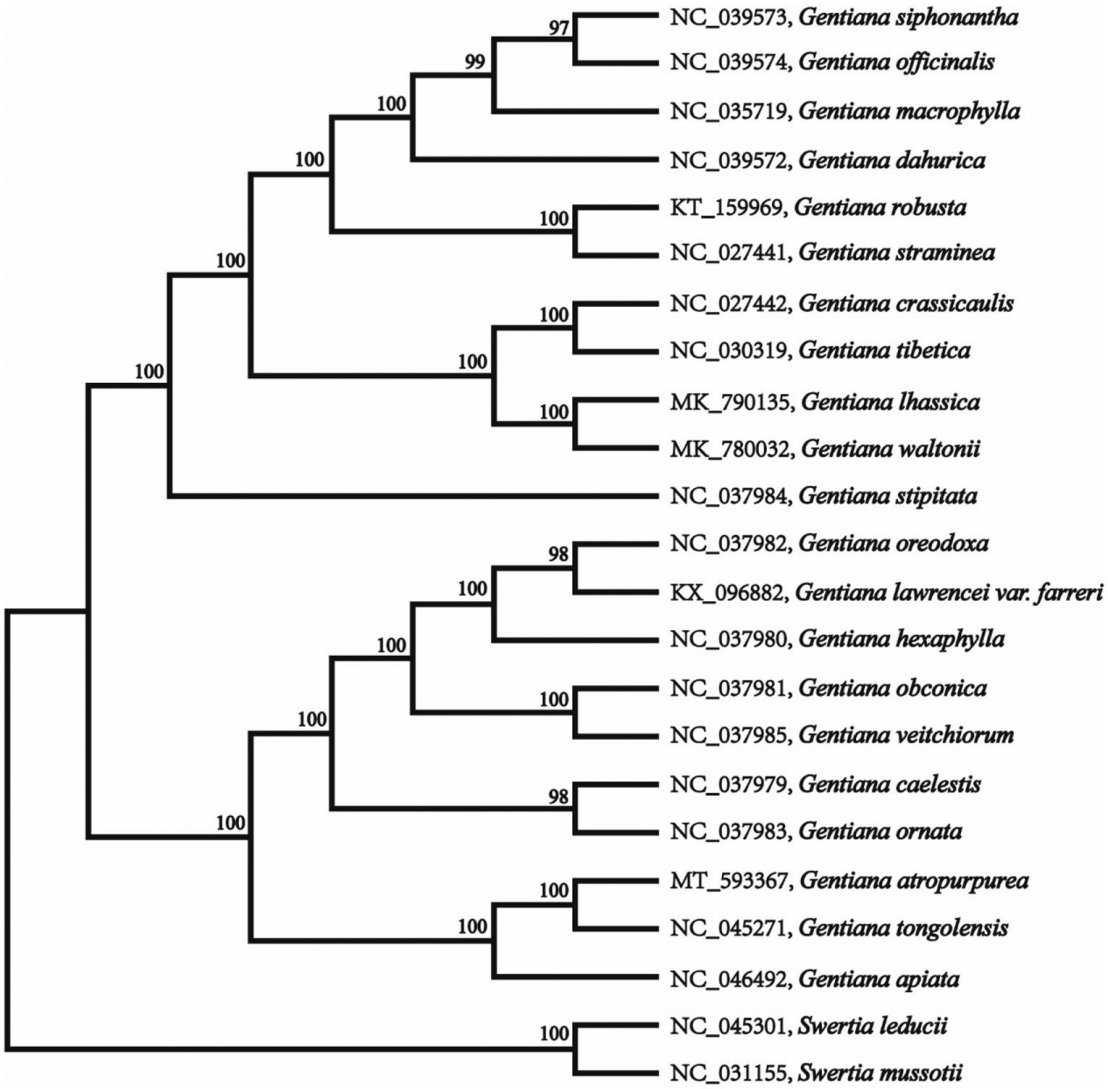
Maximum-likelihood phylogenetic tree of *G. atropurpurea* and other related species based on the complete chloroplast genome sequence.

## Data Availability

The data that support the findings of this study are available in GenBank at https://www.ncbi.nlm.nih.gov, reference number MT593367.
